# The protective effects of liguzinediol on congestive heart failure induced by myocardial infarction and its relative mechanism

**DOI:** 10.1186/s13020-020-00345-7

**Published:** 2020-06-15

**Authors:** Qi Chen, Dini Zhang, Yunhui Bi, Weiwei Zhang, Yuhan Zhang, Qinghai Meng, Yu Li, Huimin Bian

**Affiliations:** 1 School of Pharmacy, Nanjing University of Chinese Medicine, Xianlin Avenue, Qixia District, Nanjing, 210023 Jiangsu China; 2 School of Medicine and Life Sciences, Nanjing University of Chinese Medicine, Xianlin Avenue, Qixia District, Nanjing, 210023 Jiangsu China; 3 Jiangsu Key Laboratory for Pharmacology and Safety Evaluation of Chinese Materia Medica, Nanjing University of Chinese Medicine, Nanjing, 210023 China; 4 Key Laboratory on Biosafety, Nanjing Institute of Environmental Sciences, Ministry of Ecology and Environment, Nanjing, 210042 China

**Keywords:** Liguzinediol, Heart failure, Myocardial infraction, TGF-β1/Smads

## Abstract

**Background:**

Heart failure (HF) is one of the most common causes of cardiovascular diseases in the world. Currently, the drugs used to treat HF in the clinic may cause serious side effects. Liguzinediol, 2, 5-dimethyl-3, 6-dimethyl-pyrazine, is a compound synthesized after the structural modification of ligustrazine (one active ingredient of *Szechwan Lovage Rhizome*). We aimed to observe the effects of liguzinediol on preventing HF and explore the related mechanisms.

**Methods:**

The ligation of left anterior descending coronary artery was operated to established the myocardial infarction (MI) model in Sprague–Dawley rats. Cardiac functions were recorded by echocardiography and hemodynamics. The changes in the Renin–Angiotensin–Aldosterone System (RAAS), inflammation, and oxidative stress were detected by radioimmunoassay and Elisa kits. Western blot and real-time PCR were applied to determine the expressions of the TGF-β1/Smads pathway.

**Results:**

Firstly, liguzinediol enhanced the systolic and diastolic functions of the heart in MI rats. Liguzinediol improved ventricular remodeling by reducing myocardial cell necrosis, as well as reducing collagen deposition and myocardial fibrosis. Then, liguzinediol suppressed the activation of RAAS, inhibited the synthesis of pro-inflammation factors, and reduced oxidative stress. In the end, liguzinediol also down-regulated the expressions of the TGF-β1/Smads pathway.

**Conclusions:**

Liguzinediol could alleviate HF caused by MI in rats, and the protective effect was associated with the regulation of the TGF-β1/Smads pathway.
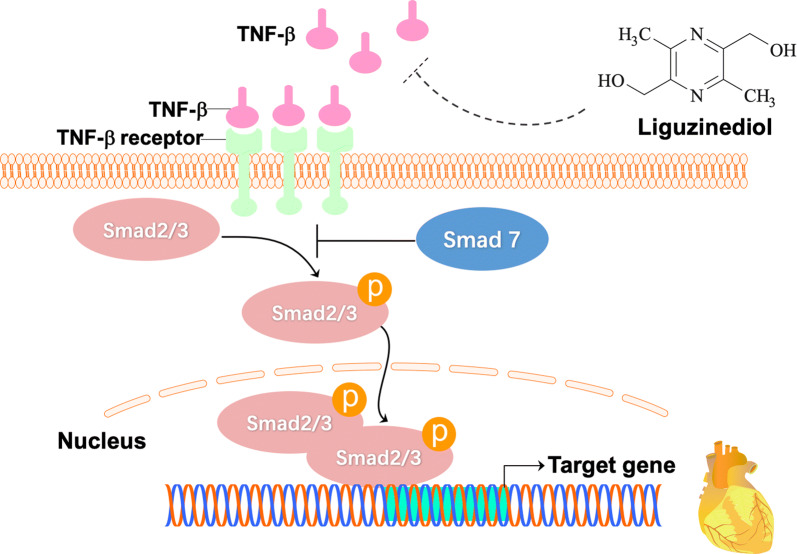

## Background

Heart failure (HF) is one of the most common causes of cardiovascular diseases with high morbidity and mortality [1]. As the average age of the population increases [2], the epidemic trend of HF also rises rapidly. Most patients with HF have a history of myocardial infarction and left ventricular remodeling [3]. The symptoms are characterized by thinning of the myocardium at the site of injury, compensatory hypertrophy of the myocardium, and enlargement of the ventricular cavity. HF has gradually become a major public health problem in China [4]. At present, clinically used anti-HF drugs are diuretics, angiotensin-converting enzyme inhibitors (ACEI), and cardiac glycosides [5]. However, these drugs can also cause serious side effects when improving the functions of the heart. For example, the safety window of cardiac glycosides is very narrow, and the dose of mild poisoning is about twice the effective therapeutic dose. In the case of myocardial ischemia and hypoxia, the poisoning dose is lower [6]. ACEI can cause transient deterioration of renal function [7], and long-term use of diuretics is prone to electrolyte disorders. Therefore, researchers have never stopped the development of new anti-HF drugs in order to improve the prognosis of HF patients while reducing toxic side effects.

**Fig. 1:**
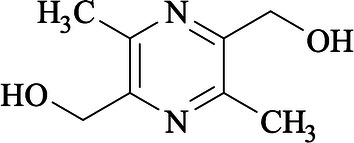
The chemical structure of liguzinediol

Liguzinediol, 2, 5-dimethyl-3, 6-dimethyl-pyrazine, is a derivative that takes ligustrazine as the lead compound for structural modification [8, 9] (Fig. 1). Liguzinediol could significantly enhance left ventricular contractility and improve the diastolic function of rat heart without arrhythmia and other adverse effects [10, 11]. Liguzinediol has the advantages of low toxicity and good water solubility, which has laid a good foundation for the research and development of non-digitalis positive inotropic drugs [12]. Our previous study revealed that liguzinediol could improve myocardial cell apoptosis in stress-induced HF [13]. But it is still unknown whether liguzinediol can improve HF caused by primary myocardial damage. In this study, we aimed to establish a model of HF caused by myocardial infarction in rats and to investigate the protective effect of liguzinediol on HF caused by myocardial infarction. As transforming growth factor-β1(TGF-β1)/Smads pathway plays an essential role in the pathogenesis of HF, we also designed to detect the effects of liguzinediol on TGF-β1/Smads pathway.

## Materials and methods

### Reagents

Liguzinediol (purity > 98%) was provided and synthesized by Professor Wei Li from Nanjing University of Chinese Medicine. The structure was elucidated and confirmed in the previous study [8]. The international patent protection of liguzinediol has been applied (No. PCT/CN2009/075100). Captopril and Digoxin were purchased from Shanghai Sine Pharmaceutical Co., Ltd (Shanghai, China). Sodium chloride, heparin, chloral hydrate, and penicillin sodium were obtained from Sigma-Aldrich (St. Louis, USA). All Elisa kits were purchased from MultiSciences (Lianke) Biotech Co., Ltd. (Hangzhou, China), and all antibodies were obtained from Abcam (Cambridge, UK).

### Myocardial infarction model and drug administration

A total of eighty male Sprague–Dawley rats (weighting 250–300 g) were obtained from Nanjing Biomedical Research Institute of Nanjing University. All rats were housed in a stable environment at a temperature of 23 ± 1 °C, the humidity of 40 ± 5%, and on a 12 h light–dark cycle. The standard diet and drinking water were supplied for rats with free access. The animal experiments were performed by the Guidelines and Policies for Animal Surgery provided by Nanjing University of Chinese Medicine, and the study was approved by the Institutional Ethics Committee of Animal Care (A171002).

Rats were intraperitoneally anesthetized with 10% chloral hydrate (300 mg/kg), and then intubated and ventilated with an automatic breathing apparatus (tidal volume: 10–12 ml; respiratory rate: 90 cycles/min; respiration ratio: 1:2). According to the previous study [14, 15], the myocardial infarction model was established by permanent left anterior descending coronary artery ligation. Briefly, the left anterior descending coronary artery was ligated at approximately 2–3 mm distal from its origin with the use of a 6–0 silk suture. Then the chest was closed immediately. A similar surgical procedure was performed in the sham group of rats without coronary artery ligation. Two weeks after surgery, ejection fraction (EF) was measured by echocardiography. Rats with EF < 55% were randomly divided into seven groups, including sham group, MI group, Captopril group (10 mg/kg), Digoxin group (0.032 mg/kg), low-liguzinediol group (5 mg/kg), medium-liguzinediol group (10 mg/kg), high-liguzinediol group (20 mg/kg). There were ten rats in each experiment group, and they were orally administrated by drugs or vehicle for 8 weeks.

### Echocardiography and hemodynamic measurements

After the anesthetic, rats underwent 2D echocardiography using an echocardiographic machine (VisualSonics Vevo2100, Canada). Echocardiographic parameters were recorded as described previously [16], including ejection fraction (EF), left ventricular fractional shortening (LVFS), LV end-systolic dimension (LVEDs) and LV end-diastolic dimension (LVEDd). Stroke volume (SV) was calculated as SV = LVEDd − LVEDs, and cardiac output (CO) was calculated as CO = SV × HR (heart rate). In addition, the hemodynamic parameters were detected by RM6240 multi-channel physiological signal acquisition and processing system (Chengdu instrument factory, China), including heart rate (HR), LV systolic pressure (LVSP), LV end-diastolic pressure (LVEDP), maximal rate of the increase of LV pressure (+dp/dt_max_) and maximal rate of the decrease of LV pressure (−dp/dt_max_), systolic blood pressure (SBP), diastolic blood pressure (DBP) and mean artery pressure (MAP).

### Biochemical assays

Blood was collected and placed at room temperature for 2 h. Serum was obtained by centrifugation at 3000 rpm for 10 min and stored at − 80 °C. Serum inflammatory cytokines tumor necrosis factor α (TNF-α), interleukin 6 (IL-6) and interleukin 1β (IL-1β) were detected by Elisa kits. The concentrations of angiotensinII (AngII), aldosterone (ALD), and plasma renin activity (PRA) were quantified by radioimmunoassay according to the manufacturer’s protocols. The levels of superoxide dismutase (SOD) and malondialdehyde (MDA) were assayed by chemichromatometry according to the directions of kits (Jiancheng Bioengineering Institute, Nanjing, China).

### Histological examination

After weighting, the hearts were fixed in 10% buffered formalin. Paraffin was used to embedded 2-mm-thick slices of the ventricle and then cut into 4 μm sections. The sections were stained by hematoxylin and eosin (H&E), and five fields were randomly selected on each section to observe [17].

### Measurement of myocardial HYP

According to the instructions of the hydroxyproline assay kit (Nanjing Jiancheng Bioengineering Institute, Nanjing, China), the content of myocardial HYP was detected by alkaline hydrolysis. Briefly, the pH of the hydrolyzed homogenate was adjusted to 6.0–6.8. Then a blank tube (containing sterile distilled water) and a standard tube (containing 5 mg/ml standard application solution) were prepared. The absorbance of each sample was analyzed at 550 nm. The content of myocardial HYP was calculated using the following formula:

$$ \begin{aligned} {\text{HYP}}\left( {\upmu{\text{g}}/{\text{mg}}} \right) & = \left( {{\text{absorbance of test tube}} - {\text{absorbance of blank tube}}} \right) \\ & \quad /\left( {{\text{absorbance of standard tube}} - {\text{absorbance of blank tube}}} \right) \\ & \quad \times {\text{content of standard tube }}\left( { 5\;\upmu{\text{g}}/{\text{ml}}} \right) \\ & \quad \times {\text{volume of hydrolysate }}\left( { 10{\text{ ml}}} \right)/{\text{wet weight of tissue }}\left( {\text{mg}} \right). \\ \end{aligned} $$

### Evaluation of collagen deposition

As mentioned before, the sections of paraffin-embedded hearts were hydrated and stained with Masson Trichrome stain (Sigma, St. Louis, MO, USA). Interstitial collagen was indicated as the percentage of blue staining. Three non-consecutive serial sections were taken from each heart, and five fields were randomly selected on each section. Also, the levels of collagen I and collagen III in serum were detected by Elisa kits.

### Ultrastructural observation

The heart tissues, about 1.0 mm × 1.0 mm × 1.0 mm in each group, were fixed with 2.5% glutaraldehyde overnight and washed three times with 0.1 mol/l (pH 7.2) phosphate buffer, then fixed with 1% osmium tetraoxide at 4 °C, washed with 0.1 mol/l phosphate buffer again, and dehydrated by ethanol at different concentrations. Epon812 resin/acetone (1:1) was applied to immerse samples for 1.5 h. Then, samples were soaked in fresh Epon812 resin for 30 min, and embedded for convergence overnight at 70 °C [18]. The tissue was cut into 60–80 nm thick slices. The ultrastructure of cardiac muscle cells was observed on transmission electron microscopy (JEM-1010, Hitachi, Tokyo, Japan).

### Western Blot analysis

The expressions of proteins involved in the TGF-β1/Smads pathway were detected. According to the instruction, the concentration of protein was identified by the BCA assay kit (Beyotime). Then 30 μg proteins were separated by 10% SDS‐PAGE and were transferred to PVDF membranes. After blocking, the primary antibodies were incubated with membranes at 4 °C overnight. Then the secondary antibody was used to incubate membranes for 90 min. In the end, the target proteins were determined with an ECL system (Millipore) and visualized by a ChemiDoc XRS system (Bio-Rad).

### Real-time polymerase chain reaction (PCR)

The mRNA levels of genes involved in the TGF-β1/Smads pathway were detected by the quantitative RT-PCR. TRIzol reagent (Invitrogen, Carlsbad) was used to isolate total RNA of J774A-1 cells. RNA was reversely transcribed into cDNA by the First Strand cDNA Synthesis kit (Thermo Fisher Scientific). The primer sequences were presented in Table 1. The Real-time PCR reaction was amplified by using ABI QuantStudio6 Q6 with AceQ qPCR SYBR^®^ Green Master Mix (Vazyme).

**Table 1:** The sequence of primers for real-time PCR

Gene name	Primer sequence (5′-3′)
TGF-β1	Sense: CCTGGAAAGGGCTCAACAC
	Antisense: CAGTTCTTCTCTGTGGAGCTGA
Smad2	Sense: CAGGACGATTAGATGAGCTTGA
	Antisense: CCCCAAATTTCAGAGCAAGT
Smad3	Sense: CCTGCCACTGTCTGCAAG
	Antisense: GCAGCAAATTCCTGGTTGTT
Smad7	Sense: ACCCCCATCACCTTAGTCG
	Antisense: AAATCCATCGGGTATCTGGA
CD105	Sense: TGGGAGGCTAGGACTCAAGA
	Antisense: TTGTGCTAGTCAAGGTGAGGAG
GAPDH	Sense: TGTTGCCATCAACGACCCCTT
	Antisense: CTCCACGACATACTCAGCA

### Statistical analysis

The data were statistically analyzed by one-way ANOVA followed by Tukey’s multiple comparison tests employing Prism (Version 6.0; GraphPad Software Inc.). Data are expressed as mean ± SD. Statistical significance was accepted at the P value of < 0.05.

## Results

### Effects of liguzinediol on echocardiographic parameters in MI rats

As shown in Table 2, before administration, the data showed that EF, LVFS, SV, and CO significantly decreased. In addition, LVEDs and LVEDd dramatically increased in MI rats. There were significant differences when compared to the sham group, which indicates that the heart failure rat model was successfully established.

**Table 2:** Effects of liguzinediol on echocardiographic parameters in MI rats

Parameters	Dose (mg/kg)	Sham	MI	Captopril	Digoxin	Liguzinediol		
				10	0.032	5	10	20
EF (%)	Before	74.88 ± 5.35	43.49 ± 10.42**	49.46 ± 9.18**	42.51 ± 4.71**	48.13 ± 9.59**	47.18 ± 10.39**	46.73 ± 9.84**
	After	74.75 ± 3.35	40.41 ± 12.21**	56.96 ± 13.57^#^**	49.37 ± 8.7**	51.9 ± 10.47^#^**	54.01 ± 8.96^#^**	54.34 ± 7.06^##^**
	D-value	− 0.13 ± 4.41	− 3.08 ± 11.41	7.50 ± 8.02^#^*	6.87 ± 7.01^#^*	3.77 ± 5.91	6.84 ± 6.77^#^*	7.60 ± 8.88^#^*
LVFS (%)	Before	45.1 ± 4.81	22.51 ± 6.21**	26.21 ± 5.65	21.95 ± 2.75**	25.43 ± 5.81**	24.83 ± 6.09**	24.5 ± 5.96**
	After	45.11 ± 3.07	21.08 ± 7.13**	31.82 ± 9.03^##^**	26.55 ± 5.65**	28.16 ± 6.47^#^**	29.48 ± 5.69^##^**	29.62 ± 4.71^##^**
	D-value	0.02 ± 4.31	− 1.42 ± 6.81	5.61 ± 5.27^#^*	4.61 ± 4.61^#^*	2.73 ± 3.56	4.65 ± 4.16^#^*	5.11 ± 5.60^#^*
LVEDd (μl)	Before	246.41 ± 33.12	369.15 ± 72.01**	329.81 ± 105.51*	341.19 ± 45.66*	343.17 ± 77.95**	323.75 ± 54.36**	319.9 ± 88.49*
	After	313.72 ± 64.81	499.06 ± 110.08**	409.29 ± 142.54	458.63 ± 48.54	450.5 ± 94.66**	445.75 ± 143.86*	425.03 ± 69.42**
	D-value	67.30 ± 69.74	129.91 ± 97.63	79.48 ± 102.45	117.44 ± 55.89	107.32 ± 60.47	122.00 ± 109.33	105.13 ± 64.13
LVEDs (μl)	Before	62.56 ± 18.13	214.87 ± 77.82**	178.92 ± 78.14**	180.27 ± 37.10**	183.64 ± 72.31**	173.44 ± 55.05**	174.88 ± 67.36**
	After	79.20 ± 24.57	306.92 ± 126.73**	186.91 ± 113.1^##^**	227.96 ± 39.82**	223.55 ± 97.08**	214.27 ± 117.18**	196.44 ± 55.52^#^**
	D-value	16.65 ± 17.50	92.05 ± 109.06*	7.99 ± 65.86	47.69 ± 33.76	39.91 ± 62.72	40.83 ± 69.18	21.55 ± 40.55
SV (μl)	Before	183.86 ± 23.50	154.28 ± 34.94*	150.89 ± 32.99*	160.91 ± 58.60*	159.53 ± 23.84*	150.31 ± 33.73*	145.01 ± 36.39*
	After	234.52 ± 42.30	192.14 ± 39.13*	222.38 ± 54.3	230.67 ± 40.01^#^	226.94 ± 37.46	231.49 ± 40.72^#^	228.59 ± 33.71^#^
	D-value	47.66 ± 62.07	37.86 ± 48.96*	71.49 ± 49.23	69.75 ± 47.24	67.41 ± 27.00	81.17 ± 52.41	83.58 ± 46.71^#^
CO (ml/min)	Before	78.39 ± 17.95	56.76 ± 13.26**	61.93 ± 19.52	68.02 ± 29.40*	62.7 ± 10.2*	60.63 ± 19.4*	64.29 ± 17.28
	After	99.69 ± 18.28**	70.41 ± 12.16**	91.11 ± 26.4^#^	92.57 ± 28.01^#^	91.32 ± 16.46^##^	95.77 ± 22.52^##^	100.41 ± 15.71^##^
	D-value	19.76 ± 24.93	13.65 ± 18.81	29.18 ± 22.59	24.54 ± 19.31	28.62 ± 13.37	35.15 ± 20.47^#^	36.11 ± 19.31^#^

Sham, sham-operated; MI, myocardial infarction; Before, before administration; After, after administration for 8 weeks; D-value = after–before; EF, ejection fraction; LVFS, left ventricular fractional shortening; LVEDd, LV end-diastolic dimension; LVEDs, LV end-systolic dimension; SV, stroke volum; CO, cardiac output, CO = SV × HR (heart rate)

Data are shown as the mean ± SD (n = 10). *P < 0.05, **P < 0.01, compared to Sham group; ^#^P < 0.05, ^##^P < 0.01, compared to MI group

At the end of administration for 8 weeks, EF and LVFS in MI rats continued to decline, LVEDs and LVEDd increased as well. Although SV and CO also increased, the difference was still significant compared with the sham group. The results implied that the heart failure of rats was not significantly improved. CO increased significantly in all administration groups. EF and LVFS were up-regulated in Captopril (10 mg/kg) and three liguzinediol (5, 10, 20 mg/kg) groups. SV improved in Digoxin (0.032 mg/kg) and two liguzinediol (10, 20 mg/kg) groups. Captopril (10 mg/kg) and liguzinediol (20 mg/kg) could also inhibit the increase of LVEDs. The original images of echocardiography were shown in Additional file 1: Figure S1.

### Effects of liguzinediol on hemodynamic parameters in MI rats

As shown in Table 3, LVSP, ± dp/dt_max_, SBP, DBP, and MAP significantly declined in MI rats, while LVEDP significantly increased when compared with the sham group. The results showed that the cardiac function of MI rats decreased obviously. After the treatment, Captopril (10 mg/kg), Digoxin (0.032 mg/kg) and liguzinediol (5, 10, 20 mg/kg) dramatically up-regulated the levels of LVSP, ± dp/dt_max_, SBP, DBP and MAP, and down-regulated LVEDP as well. It was indicated that the cardiac function of MI rats was significantly enhanced after treatment. The original images of hemodynamic parameters were shown in Additional file 2: Figure S2.

**Table 3:** Effects of liguzinediol on hemodynamic parameters in MI rats

Group	Dose (mg/kg)	LVSP (mmHg)	LVEDP (mmHg)	+dp/dt_max_ (mmHg/s)	−dp/dt_max_ (mmHg/s)	SBP (mmHg)	DBP (mmHg)	MAP (mmHg)
Sham	–	121.52 ± 11.58	12.80 ± 4.36	5486.62 ± 1274.77	− 4514.28 ± 1206.89	127.43 ± 13.73	101.40 ± 12.91	110.08 ± 12.24
MI	–	92.20 ± 9.14**	24.62 ± 11.53**	2879.57 ± 699**	− 2602.23 ± 709.79**	94.94 ± 14.45**	68.32 ± 9.91**	77.19 ± 10.94**
Captopril	10	117.70 ± 10.11^##^	11.07 ± 5.43^##^	5492.85 ± 1056.12^##^	− 4690.98 ± 407.66^##^	112.86 ± 14.92^#^*	83.65 ± 14.24^#^**	93.38 ± 13.06^##^**
Digoxin	0.032	107.94 ± 10.11^##^**	12.82 ± 4.35^##^	4334.41 ± 805.68^##^*	− 3688.32 ± 679.12^##^	117.25 ± 14.03^##^	89.02 ± 14.92^##^	98.43 ± 13.30^##^
Liguzinediol	5	112.95 ± 7.67^##^	14.50 ± 5.08^#^	4716.70 ± 761.77^##^	− 3902.99 ± 593.81^##^	117.29 ± 13.67^##^	86.93 ± 12.69^##^*	97.05 ± 12.52^##^*
	10	116.26 ± 12.91^##^	13.77 ± 4.13^#^	4870.57 ± 839.12^##^	− 4052.40 ± 914.03^##^	119.43 ± 13.13^##^	89.17 ± 11.07^##^*	99.26 ± 11.34^##^
	20	119.48 ± 7.28^##^	13.47 ± 5.15^#^	5205.27 ± 968.84^##^	− 4408.84 ± 704.99^##^	123.03 ± 14.78^##^	93.47 ± 11.70^##^	103.32 ± 12.46^##^

Sham, sham-operated; MI, myocardial infarction; LVSP, LV systolic pressure; LVEDP, LV end-diastolic pressure; +dp/dt_max_, maximal rate of the increase of LV pressure; −dp/dt_max_, maximal rate of the decrease of LV pressure; SBP, systolic blood pressure; DBP, diastolic blood pressure; MAP, mean artery pressure

Data are shown as the mean ± SD (n = 10). *P < 0.05, **P < 0.01, compared to Sham group; ^#^P < 0.05, ^##^P < 0.01, compared to MI group

### Effects of liguzinediol on HMI and LVMI in MI rats

After the hemodynamic detection, the weights of the whole heart and left ventricle were recorded. The heart mass index (HMI) and left ventricular mass index (LVMI) were also calculated. As shown in Table 4, both HMI and LVMI were significantly increased compared to the sham group, and left ventricular hypertrophy was observed in MI rats. All treatments could effectively reduce HMI and LVMI, while there was no significant difference between the MI group and the liguzinediol (5 mg/kg) group.

**Table 4:** Effects of liguzinediol on HMI and LVMI in MI rats

Group	Dose (mg/kg)	HMI (mg/g)	LVMI (mg/g)
Sham	–	2.63 ± 0.21	1.85 ± 0.21
MI	–	3.53 ± 0.30**	2.36 ± 0.43**
Captopril	10	2.88 ± 0.31^##^*	1.99 ± 0.21^#^
Digoxin	0.032	2.9 ± 0.13^##^**	2.06 ± 0.09^#^**
Liguzinediol	5	2.95 ± 0.44^##^	2.09 ± 0.31
	10	2.69 ± 0.23^##^	1.95 ± 0.18^#^
	20	2.69 ± 0.20^##^	1.92 ± 0.16^##^

Sham, sham-operated; MI, myocardial infarction; HMI, heart mass index, HMI = HM (heart mass)/BM (body mass); LVMI, left ventricular mass index, LVMI = LVM (left ventricular mass)/BM

Data are shown as the mean ± SD (n = 10). *P < 0.05, **P < 0.01, compared to Sham group; ^#^P < 0.05, ^##^P < 0.01, compared to MI group

### Effects of liguzinediol on the morphology of myocardial tissue in MI rats

**Fig. 2:**
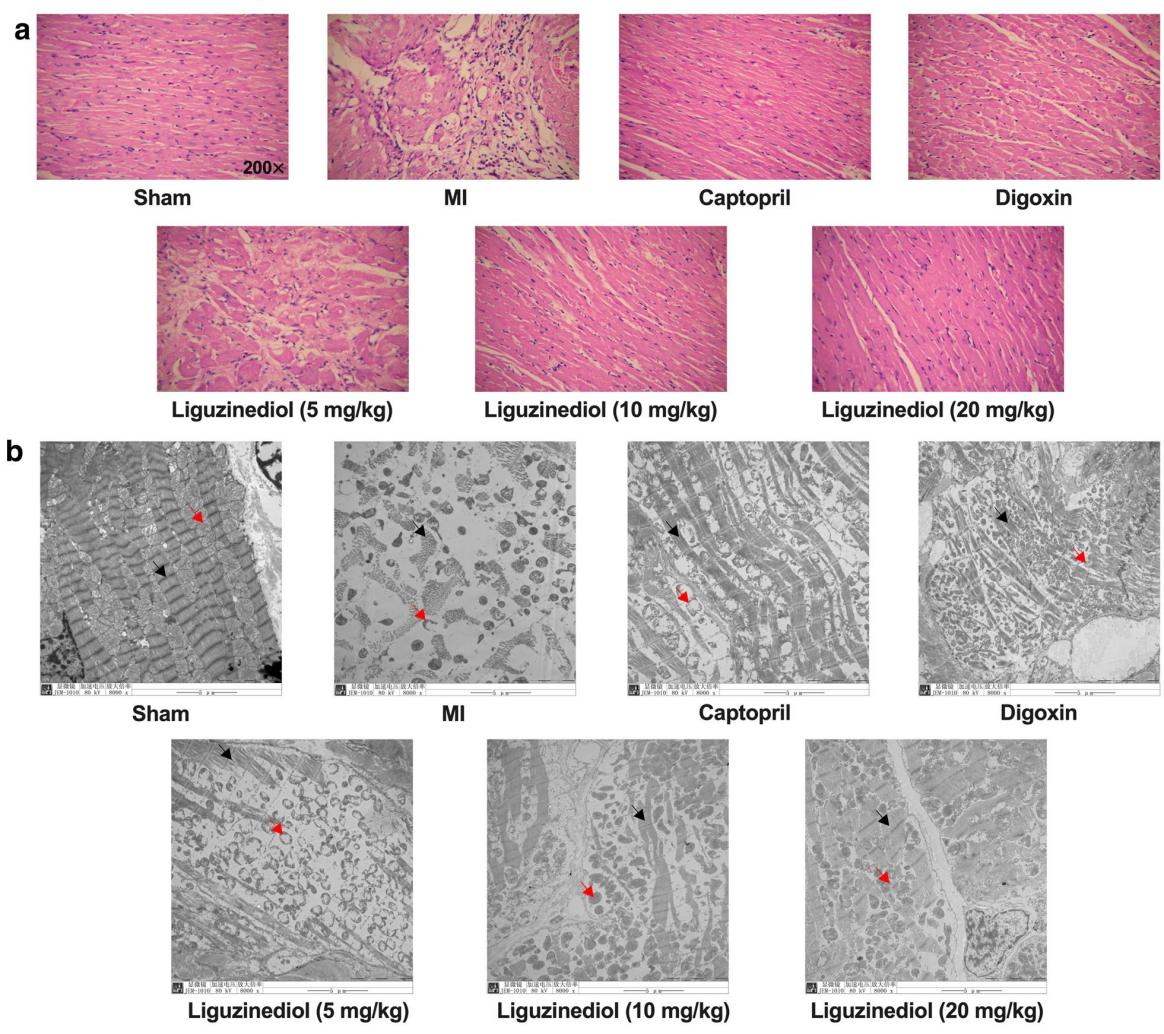
Effects of liguzinediol on of myocardial tissue in MI rats. **a** The morphology of myocardial tissue was stained by H&E (×200). **b** The ultrastructure of myocardial tissue was observed by transmission electron microscopy (×8000)

As shown in Fig. 2a, the results of H&E staining indicated that the cardiomyocytes of sham-operated rats had clear lines. No hypertrophy, atrophy, fracture, cell degeneration, necrosis, and other lesions were observed. There was no inflammatory cell infiltration in the myocardial interstitium. In MI rats, severe degeneration and necrosis of cardiomyocytes were observed. Also, severe proliferation occurred in interstitial fibrous connective tissue with mild inflammatory cell infiltration. With the treatment of Captopril (10 mg/kg), Digoxin (0.032 mg/kg), and liguzinediol (20 mg/kg), cardiomyocytes in MI rats were mildly denatured, and the cytoplasm was loose. No hyperplasia, hypertrophy, atrophy, stromal edema, and inflammatory cell infiltration were observed. In liguzinediol (5, 10 mg/kg) groups, moderate degeneration was observed in cardiomyocytes.

In addition, the ultrastructure of myocardial tissue was observed by transmission electron microscopy. As shown in Fig. 2b, the structure of mitochondrial inner and outer membranes in sham-operated rats was complete. The ridges were visible, without cavitation, and the sizes were the same. The Z and M lines of myofibril bundles are clear and arranged regularly. The nuclear structure is complete. While in MI rats, mitochondria swelled, ruptured, and formed vacuoles. The plasma membrane was partially broken, and the ridge was reduced, fractured, and scattered. Myofibrils were dissolved and disorganized. Also, the matrix density decreased and the intercellular space widened. However, all treatments could repair the mitochondrial damage in MI rats.

### Effects of liguzinediol on extracellular matrix remodeling in MI rats

**Fig. 3:**
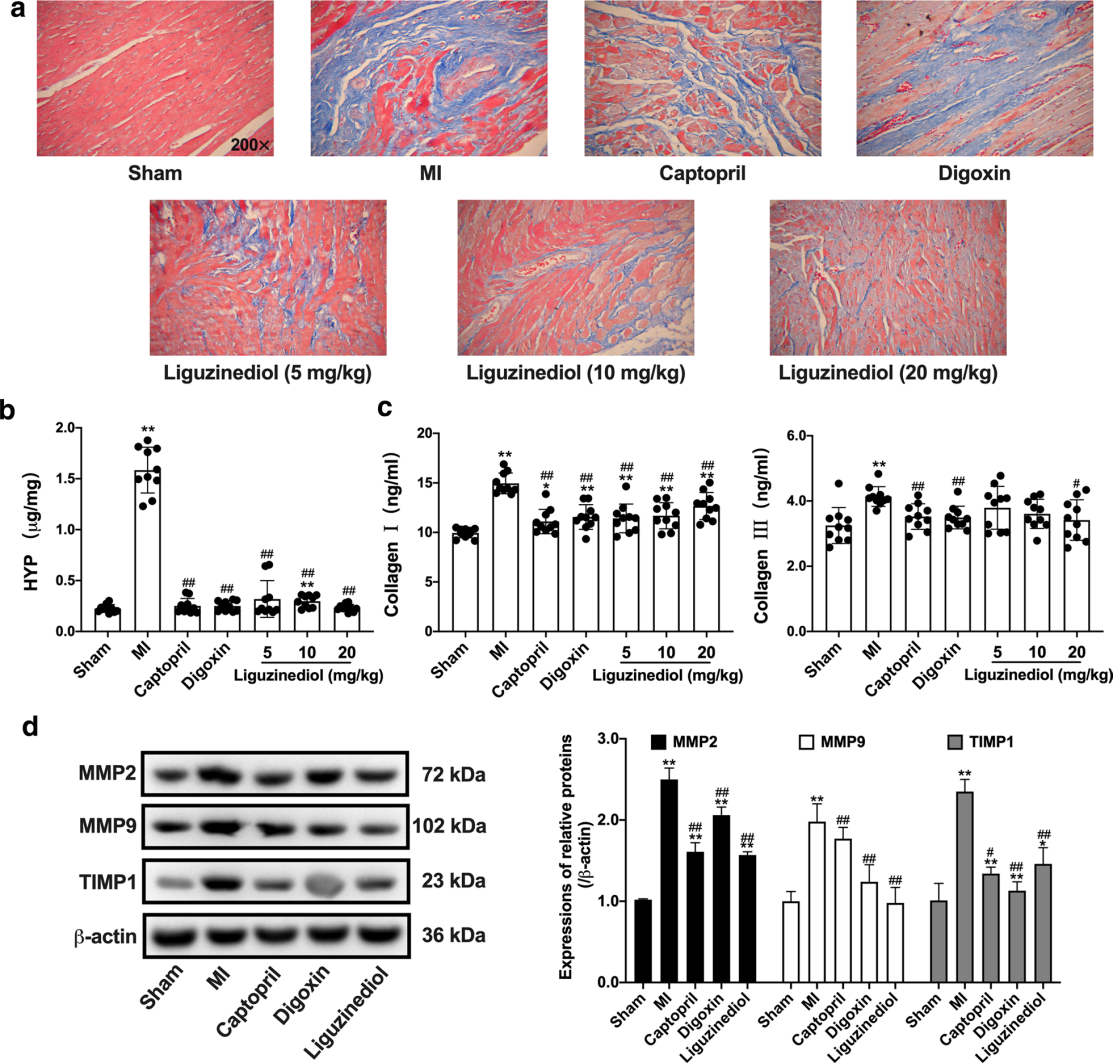
Effects of liguzinediol on extracellular matrix remodeling in MI rats. **a** The deposition of collagen fibers was stained by Masson (×200). **b** The level of HYP in myocardial tissue was detected by kits (n = 10). **c** The levels of collagen I and III in serum were detected by Elisa kits (n = 10). **d** The expressions of MMP2, MMP9 and TIMP1 in myocardial tissue were detected by western blot (n = 3). Data are shown as the mean ± SD. *P < 0.05, **P < 0.01, compared to Sham group; ^#^P < 0.05, ^##^P < 0.01, compared to MI group

The deposition of collagen fibers in the left ventricular was detected by Masson staining. As shown in Fig. 3a, the myocardial fibers in sham-operated rats were orderly arranged, and there were rare blue fibrotic tissues. In MI rats, a large number of cardiomyocytes were necrotic, and collagen fibers were significantly increased. Captopril (10 mg/kg), Digoxin (0.032 mg/kg) and liguzinediol (5, 10, 20 mg/kg) could effectively reduce collagen deposition and myocardial fibrosis.

Besides, the level of hydroxyproline (HYP) dramatically up-regulated in MI rats, and the increase was reversed by Captopril, Digoxin, and liguzinediol (Fig. 3b). Similarly, the contents of collagen I and III significantly increased in MI rats, and the deposition could be reduced by all treatments (Fig. 3c). As shown in Fig. 3d, the expressions of matrix metalloproteinases (MMPs) MMP2, MMP9, and TIMP1 were detected by western blot. Captopril, Digoxin, and liguzinediol could inhibit the declines of MMP2, MMP9, and TIMP1 in MI rats.

### Effects of liguzinediol on the Renin–Angiotensin–Aldosterone System (RAAS) in MI rats

**Fig. 4:**
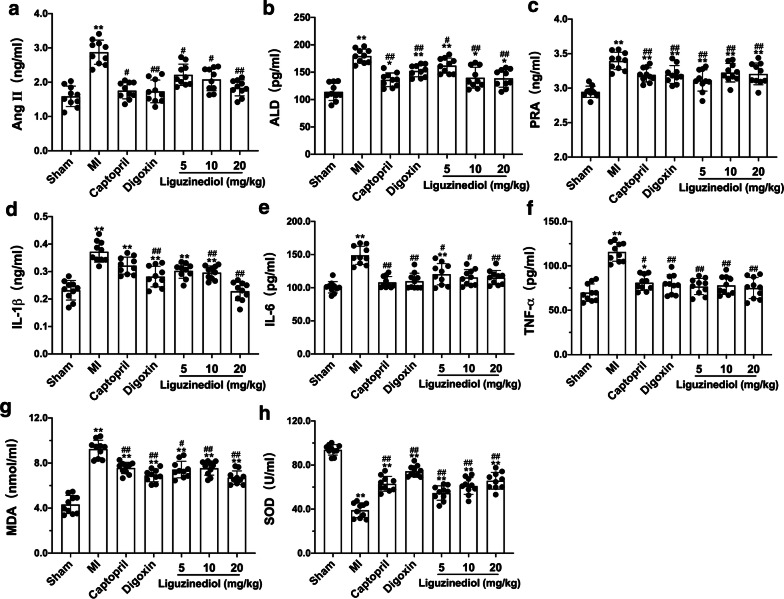
Effects of liguzinediol on RAAS, inflammation and oxidative stress in MI rats. **a**–**c** The levels of AngII, ALD and PRA in serum. **d**–**f** The levels of pro-inflammation factors IL-1β, IL-6 and TNF-α in serum. **g**, **h** The levels of MDA and SOD in LV. Data are shown as the mean ± SD (n = 10). *P < 0.05, **P < 0.01, compared to Sham group; ^#^P < 0.05, ^##^P < 0.01, compared to MI group

Activation of RAAS increases the amount of available circulating blood in the early stages of heart failure. While with the development of heart failure, the activation of RAAS can cause several harmful effects on patients. The levels of AngII, ALD, and PRA in serum were detected by Elisa kits. As shown in Fig. 4a–c, AngII, ALD, and PRA increased significantly in MI rats compared with sham-operated rats. However, all treatments down-regulated the levels of AngII, ALD, and PRA. The protective effect of liguzinediol was dose-dependent.

### Effects of liguzinediol on pro-inflammatory factors in MI rats

Inflammation plays an essential role in the pathogenesis of heart failure. Pro-inflammatory cytokines produced by cells can cause left ventricular dysfunction, ventricular remodeling, and myocardial cell necrosis to accelerate the development of heart failure further. The results showed IL-1β, IL-6, and TNF-α dramatically increased in MI rats. Compared with the MI group, all treatments could effectively reduce the level of IL-6 and TNF-α. While only Digoxin (0.032 mg/kg) and liguzinediol (10, 20 mg/kg) could significantly inhibit the increase of IL-1β in MI rats (Fig. 4d–f).

### Effects of liguzinediol on oxidative stress in MI rats

Oxidative stress maintains the balance between the pro-oxidation and anti-oxidation systems in cells. Excessive reactive oxygen species (ROS) can destroy the structure and function of myocardial cell membranes, which leads to myocardial cell necrosis. As shown in Fig. 4g, the level of MDA significantly improved in MI rats, and the enhance was reversed by all treatments. Conversely, Captopril (10 mg/kg), Digoxin (0.032 mg/kg) and liguzinediol (5, 10, 20 mg/kg) all increased the level of SOD in MI rats (Fig. 4h).

### Effects of liguzinediol on the TGF-β1/Smads pathway in MI rats

**Fig. 5:**
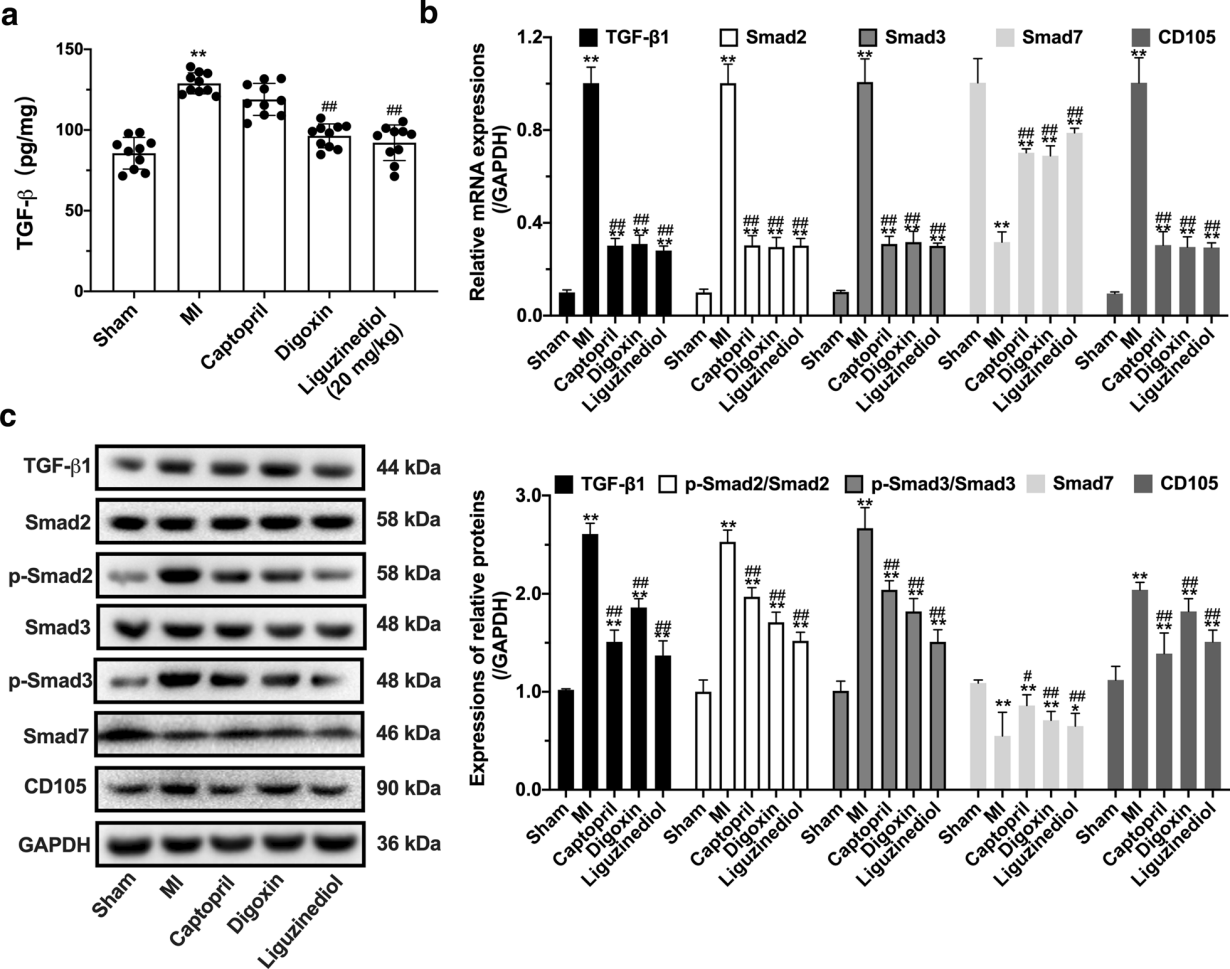
Effects of liguzinediol on TGF-β1/Smads pathway in MI rats. **a** The level of TGF-β in serum was detected by Elisa kit (n = 10). **b** Relative mRNA expressions of TGF-β1, Smad2, Smad3, Smad7 and CD105 in LV were detected by RT-PCR (n = 3). **c** Relative protein expressions of TGF-β1, Smad2, p-Smad2, Smad3, p-Smad3, Smad7 and CD105 in LV were detected by western blot (n = 3). Data are shown as the mean ± SD. *P < 0.05, **P < 0.01, compared to Sham group; ^#^P < 0.05, ^##^P < 0.01, compared to MI group

As shown in Fig. 5a, the level of TGF-β in serum significantly increased in MI rats. The treatments with Captopril (10 mg/kg), Digoxin (0.032 mg/kg) and liguzinediol (5, 10, 20 mg/kg) could effectively reduce the serum TGF-β. Then the expressions of proteins involved in the TGF-β1/Smads pathway were detected by western blot (Fig. 5c). The result showed the expressions of TGF-β1, p-Smad2, p-Smad3 and CD105 were dramatically activated in MI rats, and the increases were reversed by Captopril, Digoxin, and liguzinediol. Smad7 could inhibit the phosphorylation of Smad2 and Smad3. All treatments significantly promoted the expression of Smad7 in MI rats. Also, all treatments effectively reduced the mRNA levels of TGF-β1, Smad2, Smad3, CD105 and increased the Smad7 mRNA (Fig. 5b).

## Discussion

Heart failure has become a severe problem affecting human health worldwide, and myocardial infarction is one of the important causes of heart failure [19]. Ligation of coronary arteries is a commonly used method to establish a rat model of heart failure caused by MI [20]. In this study, we carried echocardiography combined with arterial intubation to determine the cardiac function of MI rats. Echocardiography can display the anatomical structure and ventricular wall movement of the heart in real-time, which can evaluate systolic and diastolic functions of LV [21]. The femoral artery cannula was operated to measure changes in the blood pressure of MI rats. The combination could comprehensively evaluate the changes in the cardiac function of MI rats. According to observations, EF < 55% was used as the basis for assessing the success of the HF model. Because when the EF is lower than 30%, it has been defined as severe HF, and it is meaningless to rely on drugs only [22]. The results of echocardiography and hemodynamics showed that liguziendiol could increase cardiac output and improve the systolic and diastolic functions of the heart in MI rats.

Heart failure is mainly manifested in progressive decrease of cardiac pumping function and remodeling of the ventricular cavity [23]. The core mechanism of HF is ventricular remodeling, which includes the remodeling of cardiomyocytes and extracellular matrix primarily [24]. Myocardial extracellular matrix remodeling is the result of imbalances in collagen production and degradation, as well as abnormal changes in collagen configuration and arrangement [25]. HYP is unique to collagen fibers, and the content of myocardial HYP can reflect the degree of myocardial fibrosis. The abnormal increase of HYP and collagen in myocardial tissue is the key to myocardial fibrosis [26]. The collagens present in the myocardial stroma are primarily collagen fibers type I and type III [27]. Collagen I is mainly consisted of coarse fibers with strong tension, while collagen III is fine-mesh and highly elastic. The ratio of collagen I/III can reflect myocardial stiffness [28]. Our results revealed that liguzinediol could effectively suppress the extracellular matrix remodeling and the degree of myocardial fibrosis, which enhanced myocardial compliance of MI rats. In addition, MMPs and TIMPs comprise an endogenous regulatory system that maintains the homeostasis of the extracellular matrix [29]. Several studies have reported that there was no change of MMPs activity in the early stages of compensatory cardiac hypertrophy. However, in the stage of heart failure, the increase of MMPs was positively correlated with the impairment of systolic function and the expansion of the ventricular cavity [30–32]. In this study, liguzinediol could inhibit extracellular matrix remodeling by regulating the dynamic balance of MMPs and TIMPs.

RAAS is a central system that regulates blood vessel tension, blood volume, and cardiovascular function. RAAS also plays an indispensable part in the pathogenesis of heart failure [33]. Many studies have confirmed that AngII, ALD, and PRA, the main ingredients in RAAS, significantly increased among heart failure [34–36]. Moreover, numerous clinical trials have shown that angiotensin-converting enzyme inhibitors can slow the development of heart failure, reduce morbidity and mortality of cardiovascular diseases [37, 38]. Upon RAAS is activated in MI, high levels of renin and Ang II stimulate the produce of pro-inflammatory cytokines, which may cause left ventricular dysfunction, ventricular remodeling, and myocardial cell necrosis to further accelerate the development of HF [39]. Therefore, when explaining the mechanism of HF, inflammation is an indispensable important component. During the development of MI, damage to the left ventricle promotes the synthesis and secretion of pro-inflammatory factors, including IL-1β, IL-6, and TNF-α. They can directly damage myocardial fibers, induce myocardial cell apoptosis, and cause ventricular remodeling [40, 41]. Besides, oxidative stress may be involved in the formation and development of chronic heart failure. Oxidative stress refers to the imbalance between pro-oxidation and anti-oxidant systems. Stimulated by inflammatory cytokines (such as IL-6, TNF-α), vascular endothelial cells release nitric oxide, and neutrophils produce large amounts of oxygen free radicals [42]. Excessive ROS can cause damage to the structure and function of the myocardial cell membrane, which may lead to myocardial cell necrosis and accelerate the process of HF [43]. The activity of SOD decreases in the serum of patients with HF, accompanied by an increase in MDA production, which indicates that the dynamic balance of ROS production and clearance is disrupted [44]. Based on our experimental results, we found liguzinediol could prevent HF by regulating RAAS, reducing inflammation, and inhibiting oxidative stress.

TGF-β1 is the central regulator of ventricular remodeling, which is involved in the process of myocardial fibrosis by regulating myocardial growth, myofibroblast activation, and extracellular matrix generation [45]. The intracellular TGF-β1 pathway is regulated by proteins in the Smad family. Many cardiac dysfunctions are related to changes in the TGF-β1/Smads pathway [46]. Likewise, the TGF-β1/Smads pathway has connections to RAAS, inflammation, and extracellular matrix [47]. With the treatment of liguzinediol, the expressions of the TGF-β1/Smads pathway were lowered in MI rats. It was suggested that the effect of liguzinediol on improving ventricular remodeling was related to the TGF-β1/Smads pathway. However, we did not confirm whether liguzinediol directly suppressed the expression of TGF-β1, which should be referred to in our future study. The anti-HF drugs used in the clinic can cause serious side effects. The safety window of cardiac glycosides is very narrow, ACEI can cause transient deterioration of renal function, and long-term use of diuretics is prone to electrolyte disorders. In this study, we revealed that liguzinediol exhibited protective effects on HF caused by MI. With the low toxicity and good water solubility, liguzinediol is a promising candidate for the development of new anti-HF drugs.

## Conclusion

In this study, we revealed that liguzinediol could improve cardiac function by inhibiting the activation of RAAS, suppressing the production of pro-inflammatory cytokines, and reducing oxidative stress. It may provide new insight into the molecular mechanisms for liguzinediol on preventing HF. Nevertheless, to develop liguzinediol as an eligible therapeutic agent in the clinic, more mechanisms of liguzinediol involved in preventing HF are required to be detected in future studies.

## Supplementary information

**Additional file 1: Figure S1.** The original images of echocardiography. (A) Sham group; (B) Myocardial infarction (MI) group; (C) Captopril (10 mg/kg) group; (D) Digoxin (0.032 mg/kg) group; (E) liguzinediol (5 mg/kg) group; (F) liguzinediol (10 mg/kg) group; (G) liguzinediol (20 mg/kg) group.

**Additional file 2: Figure S2.** The original images of hemodynamic parameters in MI rats. (A) Sham group; (B) Myocardial infarction (MI) group; (C) Captopril (10 mg/kg) group; (D) Digoxin (0.032 mg/kg) group; (E) liguzinediol (20 mg/kg) group.

## Data Availability

The datasets used and/or analysed during the current study are available from the corresponding author on reasonable request.

## Abbreviations

ACEI: Angiotensin-converting enzyme inhibitors
ALD: Aldosterone
AngII: AngiotensinII
CO: Cardiac output
DBP: Diastolic blood pressure
+dp/dt_max_: Maximal rate of the increase of LV pressure
−dp/dt_max_: Maximal rate of the decrease of LV pressure
EF: Ejection fraction
H&E: Hematoxylin and eosin
HF: Heart failure
HMI: Heart mass index
HR: Heart rate
HYP: Hydroxyproline
IL-1β: Interleukin-1β
IL-6: Interleukin-6
LV: Left ventricular
LVEDd: LV end-systolic dimension
LVEDs: LV end-systolic dimension
LVEDP: LV end-diastolic pressure
LVFS: LV fractional shortening
LVMI: Left ventricular mass index
LVSP: LV systolic pressure
MAP: Mean artery pressure
MDA: Malondialdehyde
MI: Myocardial infarction
MMPs: Matrix metalloproteinases
PRA: Plasma renin activity
RAAS: Renin–Angiotensin–Aldosterone System
ROS: Reactive oxygen species
RT-PCR: Real-time polymerase chain reaction
SBP: Systolic blood pressure
SOD: Superoxide dismutase
SV: Stroke volume
TGF-β1: Transforming growth factor-β1
TNF-α: Tumor necrosis factor-α
