# Supplementary figures and images for: The protective effects of liguzinediol on congestive heart failure induced by myocardial infarction and its relative mechanism
Source: Chin Med. 2020 Jun 15;15:63. doi: 10.1186/s13020-020-00345-7 (PMC7296683; doi:10.1186/s13020-020-00345-7)

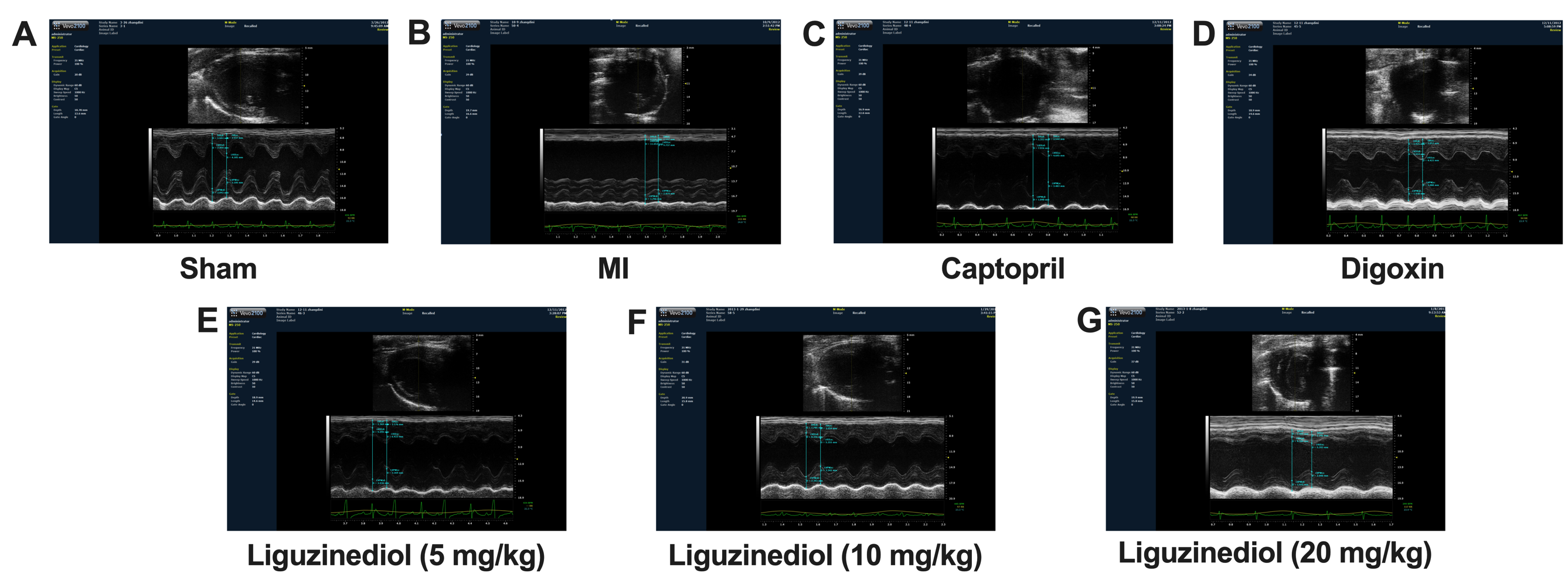

Supplement: Supplementary file 1 — Additional file 1: Figure S1. The original images of echocardiography. (A) Sham group; (B) Myocardial infarction (MI) group; (C) Captopril (10 mg/kg) group; (D) Digoxin (0.032 mg/kg) group; (E) liguzinediol (5 mg/kg) group; (F) liguzinediol (10 mg/kg) group; (G) liguzinediol (20 mg/kg) group. [file 13020_2020_345_MOESM1_ESM.tiff]

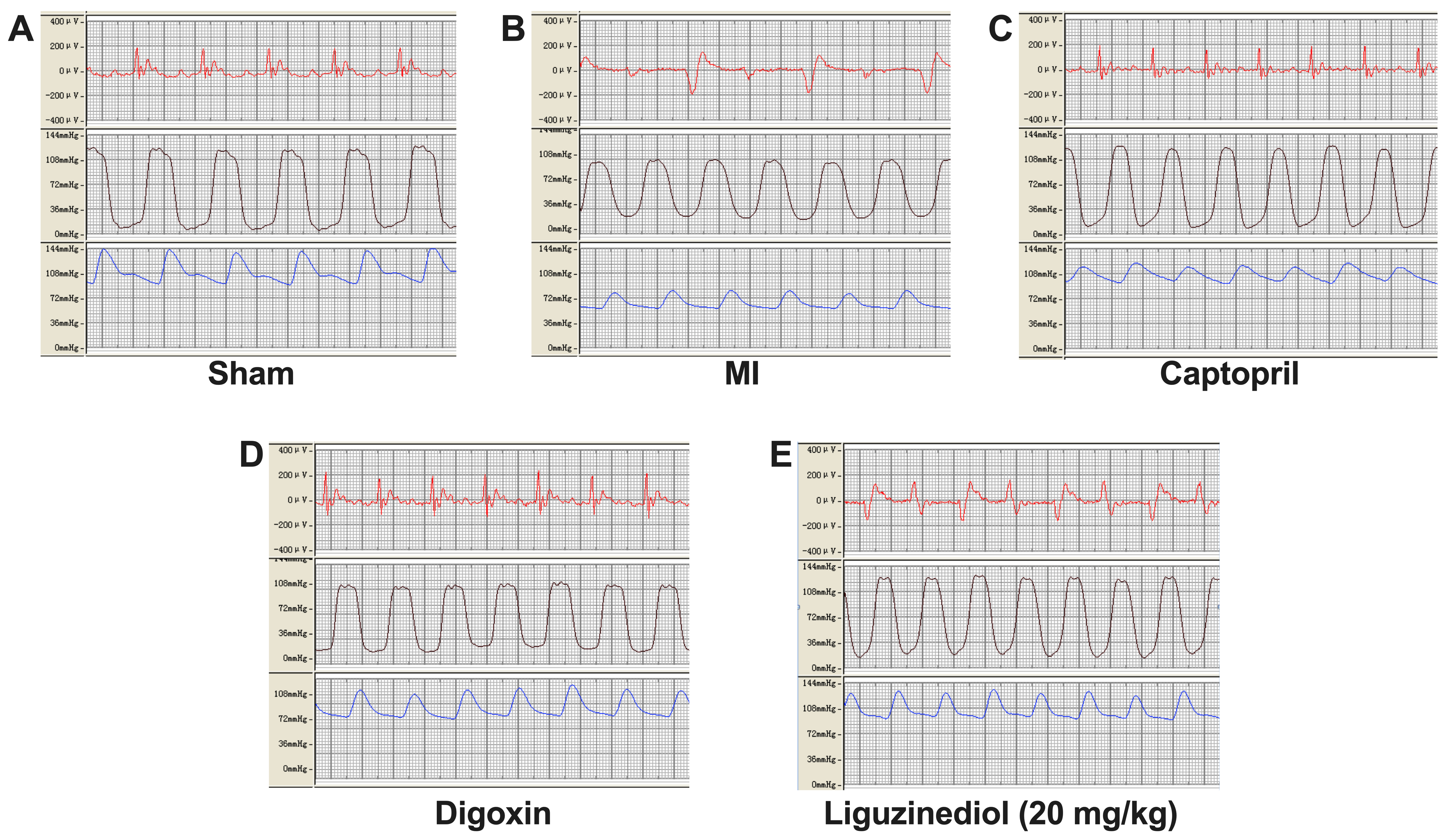

Supplement: Supplementary file 2 — Additional file 2: Figure S2. The original images of hemodynamic parameters in MI rats. (A) Sham group; (B) Myocardial infarction (MI) group; (C) Captopril (10 mg/kg) group; (D) Digoxin (0.032 mg/kg) group; (E) liguzinediol (20 mg/kg) group. [file 13020_2020_345_MOESM2_ESM.tiff]
